# Risk factors of postoperative cerebral hyperperfusion syndrome and its relationship with clinical prognosis in adult patients with moyamoya disease

**DOI:** 10.1186/s41016-023-00321-8

**Published:** 2023-04-03

**Authors:** Zhiyong Shi, Lingyun Wu, Yi Wang, Huasheng Zhang, Yongbo Yang, Chunhua Hang

**Affiliations:** Department of Neurosurgery, Nanjing Drum Tower Hospital, The Affiliated Hospital of Nanjing University Medical School, No. 321 Zhongshan Road, Nanjing, Jiangsu Province MN 210008 People’s Republic of China

**Keywords:** Moyamoya disease, Combined cerebral revascularization, Cerebral hyperperfusion syndrome, Risk factors, Clinical prognosis

## Abstract

**Background:**

To investigate the incidence, risk factors, and clinical prognosis of cerebral hyperperfusion syndrome (CHS) after superficial temporal artery-middle cerebral artery anastomosis combined with encephalo-duro-arterio-synangiosis (STA-MCA/EDAS) in adult patients with moyamoya disease (MMD).

**Methods:**

The clinical data of 160 adult patients with MMD treated by STA-MCA/EDAS from January 2016 to January 2017 were retrospectively analyzed. According to CHS diagnosis, MMD patients were divided into CHS and non-CHS group. Univariate and multivariate analysis of risk factors and Kaplan-Meier curve of stroke-free survival for CHS were performed.

**Results:**

A total of 12 patients (7.5%) developed postoperative CHS, of which 4 patients (2.5%) presented with cerebral hemorrhage. Univariate and multivariate analysis showed moyamoya vessel on the surgical hemisphere (*OR* = 3.04, 95% *CI* = 1.02–9.03, *P* = 0.046) and left operated hemisphere (*OR* = 5.16, 95% *CI* = 1.09–21.34, *P* = 0.041) were independent risk factors for CHS. The other variables, such as age, gender, presentation, hypertension, diabetes, smoking, mean mRS score on admission, modified Suzuki stage and pre-infarction stage on surgical hemisphere, and bypass patency, had no association with postoperative CHS (*P* > 0.05). At final follow-up with average 38 months, there were 18 out of 133 patients (13.5%, 4.91% per person year) presented with newly developed complications. There was no significant difference between newly developed complications, mean mRS scores, and Kaplan-Meier curve of stroke-free survival in patients with and without CHS (*P* > 0.05).

**Conclusion:**

The concentration of moyamoya vessels and left operated hemisphere was independent risk factors for CHS, which could not affect the clinical prognosis if treated timely and properly. The current study offers a new perspective of moyamoya vessels and supporting data for choosing MMD candidates on cerebral revascularization.

## Background

Moyamoya disease (MMD) was characterized by bilateral, chronic, progressive stenosis or even occlusion at terminal portion of the internal carotid artery (ICA), middle cerebral artery (MCA), anterior cerebral artery (ACA), and formation of moyamoya vessels at base of brain [1, 2]. Surgical revascularization was the best treatment for most patients with MMD, which included direct, indirect, and combined revascularization to convert blood from extracranial arteries to intracranial arteries, thereby changing cerebral hemodynamic status and improving patient’s prognosis [3–6]. However, cerebral hyperperfusion syndrome (CHS) was a common complication after direct or combined cerebral revascularization manifesting with unilateral headache, epilepsy, aphasia, and motor and sensory disorders [7]. Cerebral hemorrhage caused by CHS was fatal complication if not treated in time, with the percentage from 3.3 to 6.6% [8–11]. Zhang et al. reported that direct anastomoses of recipient artery with antegrade hemodynamics source from MCA was associated with postoperative CHS [12]. In addition, adult-onset or hemorrhagic-onset patients were also reported to have significantly higher risk for symptomatic hyperperfusion [13]. Besides, the mRS score on admission and ischemic presentation before surgery were also independent risk factors of postoperative CHS [14]. The mechanism underlying CHS in patients with MMD still remained unclear. This research aimed to explore the incidence, risk factors, and clinical prognosis of CHS after superficial temporal artery-middle cerebral artery bypass combined with encephalo-duro-arterio-synangiosis (STA-MCA/EDAS) in adult patients with MMD.

## Methods

### Patient selection

This research was approved by the Ethics Committee of Nanjing Drum Tower Hospital, and informed consent was obtained. The admission criteria of patients with MMD were as follows: (1) diagnosed with MMD according to the guidelines proposed by the Ministry of Health and Welfare of Japan [15], (2) aged ≥ 18 years old, (3) treated by combined cerebral revascularization (STA-MCA/EDAS), (4) underwent both MR angiography (MRA) and CT perfusion (CTP) scans before and 1 week after surgery, and (5) underwent postoperative MRI was performed to exclude ischemia. Exclusion criteria were as follows: (1) patients with moyamoya syndrome (MMS) [16], (2) patients with age < 18 years old, (3) patients with conservative treatment and indirect bypass alone, and (4) patients diagnosed with postoperative ischemia-related complications by diffusion-weighted imaging (DWI) sequence.

The clinical data of 160 adult MMD patients treated by STA-MCA/EDAS in Nanjing Drum Tower Hospital from January 2016 to January 2017 was collected and analyzed. According to CHS diagnosis, adult MMD patients treated by STA-MCA/EDAS were divided into non-CHS group and CHS group.

### Imaging protocol

**Fig. 1:**
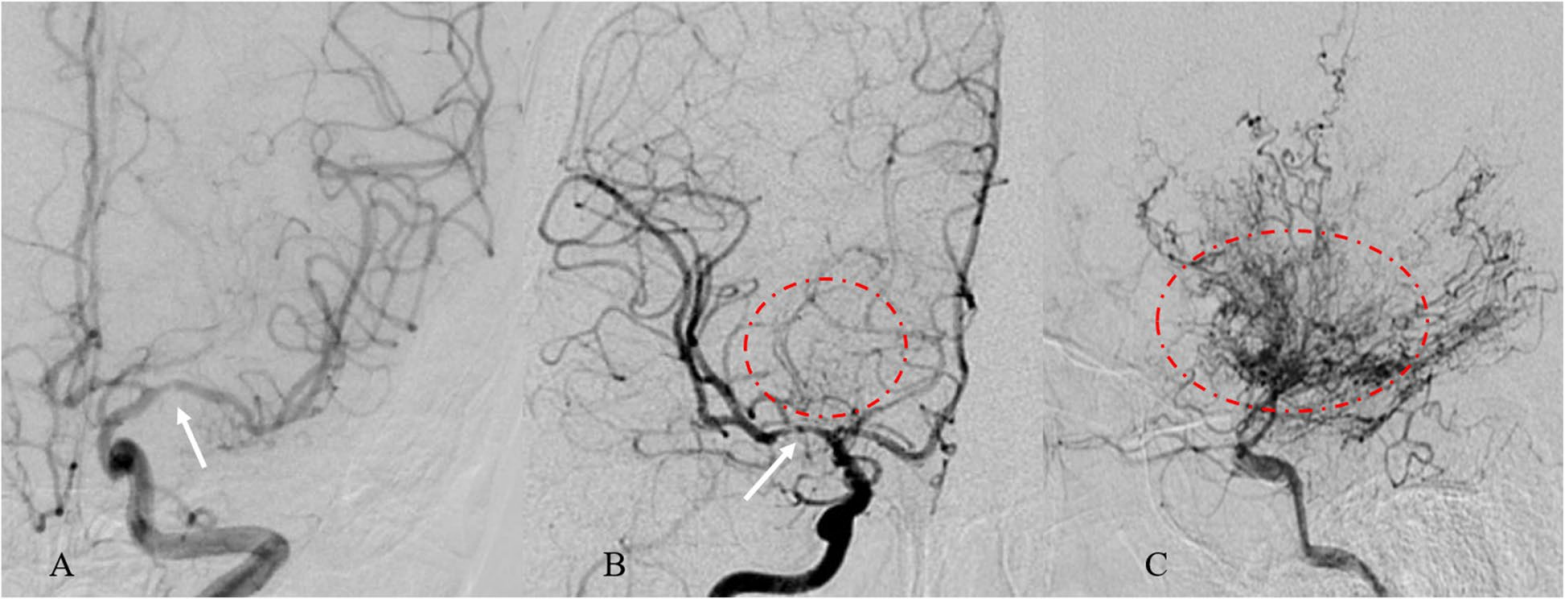
Illustration of moyamoya vessels concentration. **A** Mild stenosis of M1 segment (arrow) and no moyamoya vessels formed. **B** Severe stenosis of M1 segment (arrow) and smoke vessels formed but sparsely (circle). **C** Extensive moyamoya vessels formed and expanded in all directions (circle)

Based on the Suzuki stage (SS) of surgical hemisphere, modified Suzuki stage was performed, with SS I and II in early stage, SS III and IV in middle stage, and SS V and VI in advanced stage [17]. Based on concentration of moyamoya vessels arising from surgical hemisphere, patients were divided into “none group” (no obvious puff smoke formation), “sparse group” (smoke vessels formed at base of brain but sparsely), and “dense group” (extensive moyamoya vessels formed and expanded in all directions) [18] (Fig. 1).

In addition, CTP parameters included cerebral blood volume (CBV), cerebral blood flow (CBF), mean transmit time (MTT), and time to peak (TTP). According to CTP parameters of operative hemisphere, cerebral pre-infarction stage was divided into stage 1 (TTP delayed, MTT, CBF, and CBV were normal), stage 2 (TTP, MTT delayed, CBF and CBV were normal or slightly increased), stage 3 (TTP, MTT delayed, CBF decreased, and CBV was normal or slightly decreased), and stage 4 (TTP and MTT delayed, and CBF and CBV were decreased) [19] (Table 1).

**Table 1:** Description of pre-infarction stage of hemisphere based on CTP. Nc, not changed

	TTP	MTT	CBV	CBF
I	delayed	nc	nc	nc
II	delayed	delayed	nc	nc
III	delayed	delayed	increased	nc
IV	delayed	delayed	decreased	decreased

### Postoperative CHS

**Fig. 2:**
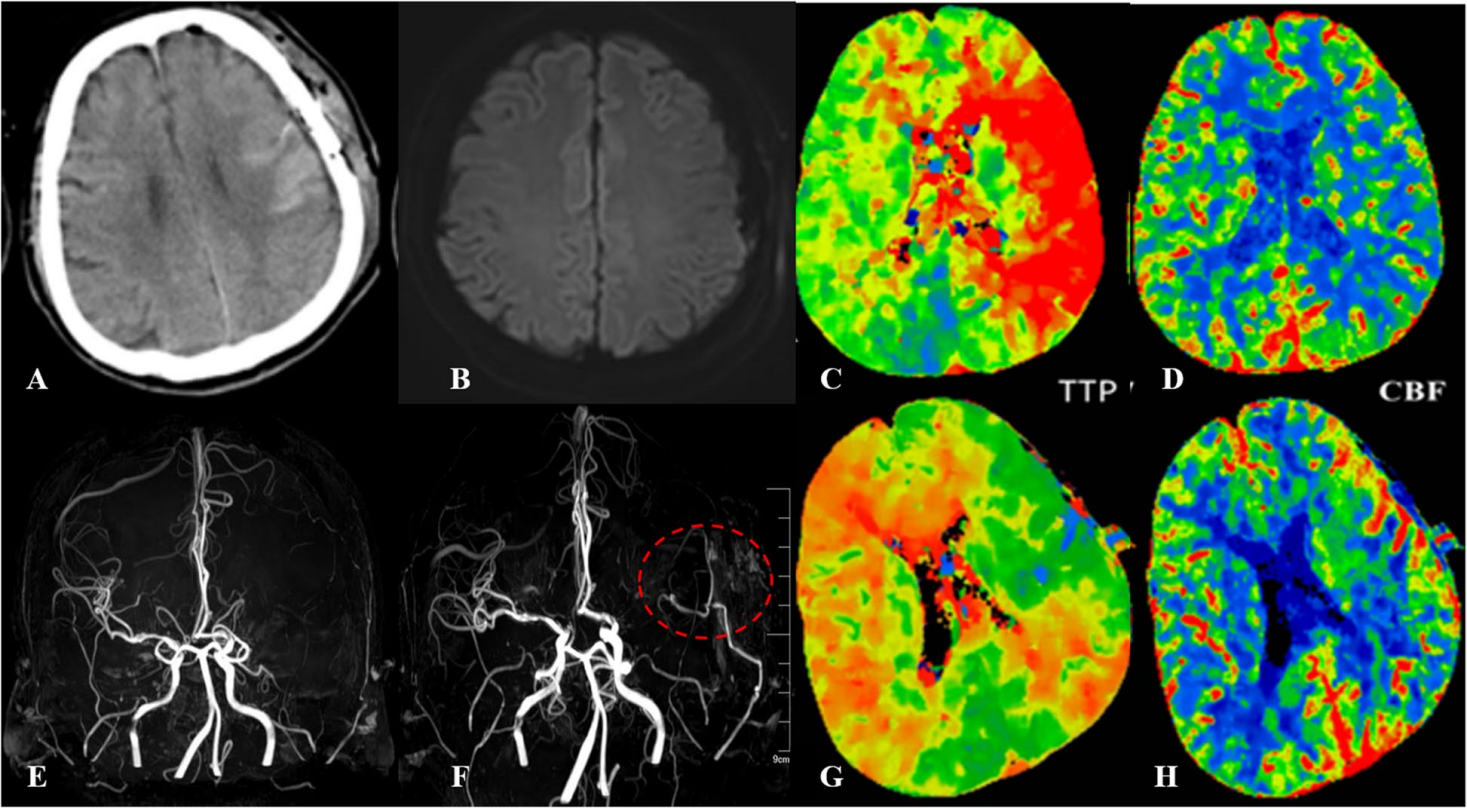
Illustration of a 50-year-old patient with CHS. This MMD patient presented with speech dysfunction and right limb paresthesia and was treated with combined bypass surgery on left hemisphere. On 2 days after surgery, he suffered from aphasia, hemiplegia and emergency CT found high-density sign around operative area (**A**), and no infarcted sign on DWI image (**B**). When compared with MRA before surgery (**E**), bypass artery was enlarged (**F**, circle). Hemispheric perfusion was improved after revascularization, with TTP decreased (**C** and **G**) and CBF increased (**D** and **H**). After treatment of blood control, fluid dehydration, and antiepileptic drug, he recovered gradually and discharged with no symptoms

CHS was defined as a cluster of clinical symptoms that occurred after STA-MCA/EDAS bypass, including ipsilateral headache, epilepsy, aphasia, motor or sensory disturbances, and other neurological impairments [20, 21]. For cases complained of discomfort after surgery, bypass pulsation palpation in front of the ear, and the emergent MRA/CTP examinations, should be performed to determine the cause of discomfort. CHS was mainly diagnosed with the following radiological items: (1) the presence of significant regional CBF increase around the anastomosis site (qualitative observation of an intense focal increase in pre-infarction stage); (2) apparent visualization of STA-MCA bypass by MRA; and (3) postoperative MRI, including diffusion-weighted imaging (DWI), were performed to exclude possible ischemic pathology (Fig. 2). Most of these symptoms occurred at 2 days after direct revascularization and might resolve in 2 weeks without permanent brain injury. Patients with CHS were treated with strict blood pressure control, appropriate rehydration, preventive use of anti-epileptic drugs, and others.

### Clinical follow-up

All MMD patients were followed up at minimal 12 months after surgery. In addition, new onset of neurological-related complications was also recorded, including transient ischemic attack (TIA), cerebral infarction, and brain hemorrhage. At discharged and final follow-up, mRS score of patients was evaluated.

### Statistical analysis

We used *χ*^2^ test for categorical variables and independent sample *t*-test for continuous variables with normal distribution. Rank-sum test was used for rank or skewed distribution variables. Multivariate logistic regression was used to determine the risk factors of postoperative CHS. Stroke-free survival analysis was performed using Kaplan-Meier curves, with comparisons made using log-rank statistics. Statistical analysis was performed with SPSS 22.0 (IBM, USA). A significant level was set at *P* < 0.05.

## Results

### General patient characteristics

Of 160 adult patients with MMD, 77 males and 83 females met the inclusion criteria; age ranged from 19 to 60 years old, mean 40 years old; 60 patients presented with hemorrhage, and 100 patients had ischemia. The baseline characteristic of adult patients with MMD were listed in Table 2. In 12 (7.5%) of 160 patients, postoperative CHS developed, including ipsilateral headache in 3 cases, cerebral hemorrhage in 4 cases, dysarthria in 3 cases, and motor disorder and seizure in 1 case, respectively.

**Table 2:** Characteristics of patients with and without postoperative CHS after combined cerebral revascularization (*n*, %)

Characteristic	Postoperative CHS		*P*-value		*OR* (95% *CI*)
	Absent (*n* = 148)	Present (*n* = 12)	Univariate	Multivariate	
Age (years old)	40.05 ± 9.34	38.42 ± 11.21	0.567		
Gender (male)	71 (48%)	6 (50%)	1		
Clinical type			0.537		
Ischemic	91 (61.5%)	9 (75%)			
Hemorrhagic	57 (38.5%)	3 (25%)			
Hypertension	55 (37.2%)	5 (41.7%)	0.764		
Diabetes	21 (14.2%)	0(0%)	0.369		
Smoking	19 (12.8%)	2 (16.7%)	0.659		
Mean mRS on admission	1.16 ± 0.61	1.25 ± 0.62	0.609		
Suzuki stage			0.103		
Early	21 (14.2%)	0 (0%)			
Moderate	78 (52.7%)	10(83.3%)			
Advanced	49 (33.1%)	2 (16.7%)			
Moyamoya vessels			**0.032**^**a**^	**0.046**^**a**^	3.04 (1.02–9.03)
None	26 (17.6%)	1 (8.3%)			
Sparse	66 (44.6%)	2 (16.7%)			
Dense	56 (37.8%)	9 (75%)			
Pre-infarction stage			0.070	0.368	1.28 (0.75–2.19)
I	12 (8.1%)	0 (0%)			
II	55 (37.2%)	4 (33.3%)			
III	65 (43.9%)	4 (33.4%)			
IV	16 (10.8%)	4 (33.5%)			
Operative side (left) 86 (58.1%)		11 (91.7%)	**0.029**^**a**^	0.041^a^	5.16 (1.09–21.34)
Bypass patency	147 (99.3%)	12 (100%)	1		

*CHS* cerebral hyperperfusion syndrome, *mRS* modified Rankin score

^a^*P* < 0.05

For cases with postoperative CHS, dense, sparse, and none moyamoya vessels were observed in 9, 2, and 1 cases, respectively. Onset time of postoperative CHS ranged from postsurgical 1 to 5 days. Onset symptoms included hemorrhage in 4 cases, dysarthria in 3 cases, headache in 3 cases, motor disorder in 1 case, and seizure in 1 case. For cases with postoperative hemorrhage, hematoma evacuation surgery was performed in 3 cases. Hematoma had a predilection for the cortex around anastomosis site, where we suspected fragile, ruptured leptomeningeal artery was the criminal. Extensively strict blood pressure control and proper rehydration were performed in 9 cases, which were particularly important to balance the risk of hyperperfusion caused by a sudden increase of blood flow and of ischemia caused by insufficient blood supply after surgery. The other 3 cases with cerebral hemorrhage were transferred for extensive care. Eight (75%) patients with CHS could recover without severe sequelae when discharged (Table 3).

**Table 3:** Detailed information of adult MMD patients with postoperative CHS

No.	Sex	Age (yrs)	HT	Presentation	Smoking	Ss	Mv	Pc	Sx	OT	Treatment	Discharged mRs
1	M	58	Yes	TIA	No	4	D	4→3	Headache	P1	BP control	1
2	M	44	No	Headache	No	4	D	2→2	Dysarthria	P2	BP control	1
3	M	24	No	TA	No	3	D	4→2	Hemorrhage	P2	HE→ICU	1
4	M	41	No	Bleeding	No	3	D	2→2	Headache	P3	BP control	1
5	F	54	Yes	TIA	Yes	6	N	2→2	Motor disorder	P1	BP control	2
6	F	33	Yes	Infarction	No	3	D	3→2	Headache	P3	BP control	1
7	M	30	No	Infarction	No	4	D	4→3	Hemorrhage	P3	HE→ICU	2
8	F	21	No	TIA	No	3	D	2→2	Dysarthria	P2	BP control	2
9	F	35	No	Infarction	No	5	S	3→2	Seizure	P5	BP control	1
10	F	46	Yes	Infarction	Yes	4	D	3→1	Hemorrhage	P1	HE→ICU	2
11	M	41	Yes	Bleeding	Yes	3	D	4→2	Dysarthria	P2	BP control	1
12	F	34	No	Infarction	No	4	S	3→2	Hemorrhage	P3	BP control	2

*HT* hypertension, *Ss* Suzuki stage, *Mv* moyamoya, *D* dense, *N* none, *S* sparse, *Pc* perfusion change (pre-operation → post-operation), *Sx* symptom, *OT* onset time, *BP* blood pressure, *HE* hematoma evacuation

### Univariate and multivariate analysis for CHS

According to univariate analysis, moyamoya vessels and operative hemisphere were significantly associated with postoperative CHS (*P* < 0.05). The other variables, such as age, gender, presentation, hypertension, diabetes, smoking, mean mRS score on admission, modified Suzuki stage and pre-infarction stage on surgical hemisphere, and bypass patency, had no association with postoperative CHS (*P* > 0.05). Multivariate regression analysis indicated that moyamoya vessels (*OR* = 3.04, 95% *CI* = 1.02–9.03, *P* = 0.046) and left operated hemisphere (*OR* = 5.16, 95% *CI* = 1.09–21.34, *P* = 0.041) were statistically associated with postoperative CHS (Table 2).

### Follow-up outcome

**Fig. 3:**
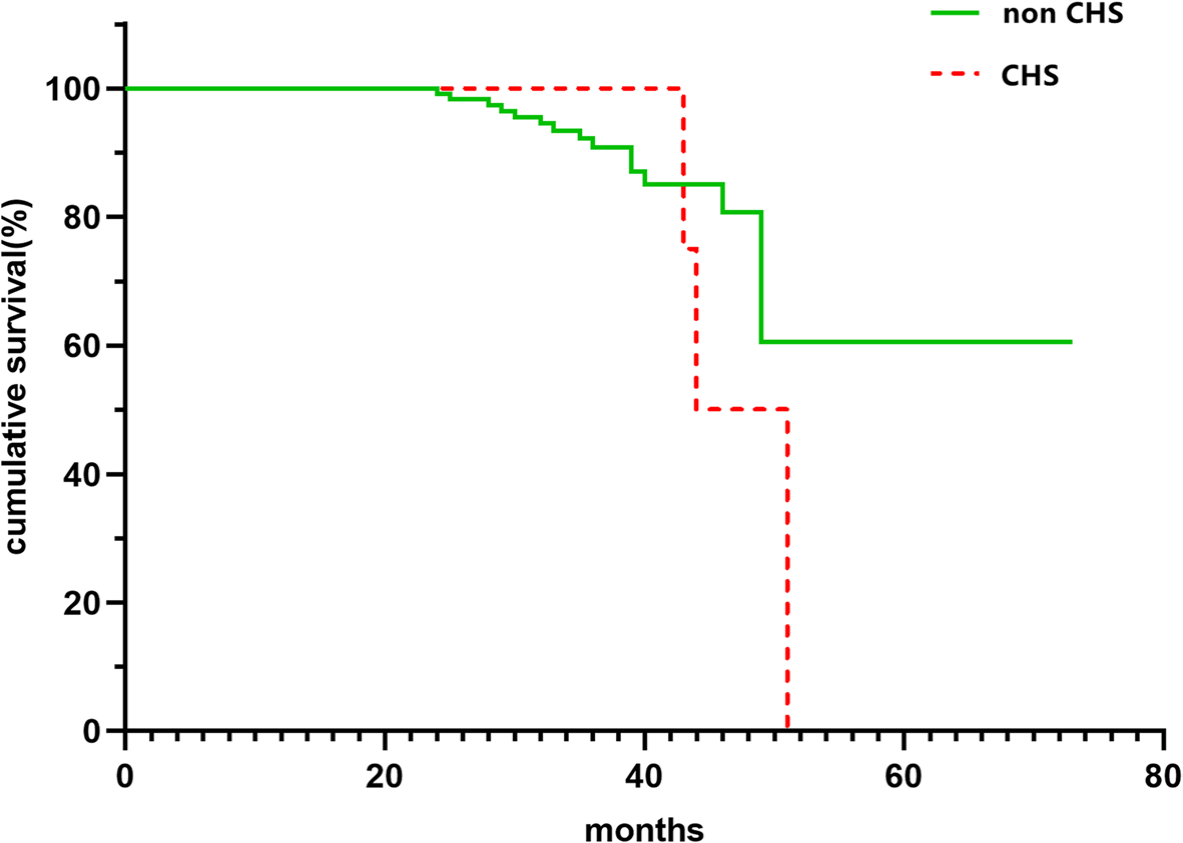
Kaplan-Meier cumulative hazard curve for stroke-free surgical when comparing patients in CHS group and non-CHS group

When discharged, there was significant difference of mean mRS score between patients with and without CHS (1.42 ± 0.52 vs 0.99 ± 0.68, *P* = 0.036) (Table 4). However, at final follow-up visits with an average of 38 months, 133 cases were followed up. A total of 18 cases (13.5%, 4.91% per person year) presented with newly developed neurological complications, of which 15 cases in the non-CHS group and 3 cases in CHS group. There was no significant difference in newly developed complications between two subgroups (*P* > 0.05). Moreover, there was no significant in the mean mRS score difference between patients in CHS group and non-CHS group during follow-up (*P* = 0.210) (Table 4). In addition, there had no statistically significance in the Kaplan-Meier curve analysis of stroke-free survival between the patients with and without CHS (Fig. 3).

**Table 4:** Comparison of clinical follow-up outcome for MMD cases with and without postoperative CHS

Characteristic	Postoperative CHS		*t*/*χ*^2^	*P*-value
	Absent (*n* = 122)	Present (*n* = 11)		
Mean mRS at discharge	0.99 ± 0.68	1.42 ± 0.52	−2.013	0.036*
Follow-up (months)	37.97 ± 12.35	43.33 ± 12.26	−1.449	0.149
New complications	15 (12.3%)	3 (27.3%)	1.934	0.171
TIA	8 (6.56%)	2 (18.18%)		
Infarction	6 (4.92%)	1 (9.09%)		
Hemorrhage	1 (0.82%)	0 (0%)		
Mean mRS at follow-up	0.91 ± 0.68	1.36 ± 1.12	−1.335	0.211

^*^P < 0.05

## Discussion

Cerebral revascularization was a recognized treatment for patients with MMD, which could prevent the recurrence of cerebral ischemia or hemorrhage and improve the prognosis of patients [22–25]. According to literature published, postoperative CHS was a common complication after direct or combined cerebral revascularization, and the incidence was from 6.7 to 38.2% [8, 26–28]. In contrast to previous research, the incidence of postoperative CHS in this study was about 7.5%, which was consistent with previous reports.

CHS was mostly caused by the contradiction between the impaired cerebrovascular autoregulation of intracranial arteries, increased vascular permeability, and the sudden increase of blood input from bypass artery [29]. Other researchers also reported that postoperative CHS for patients with MMD was associated with unstable status of cerebral hemodynamics after combined or direct bypass revascularization, causing local hyperperfusion and hemispheric hypoperfusion [30]. Adult, hemorrhagic-onset patients and mRS score on admission were associated with postoperative CHS [13, 14]. In this report, the left hemisphere operated was significantly associated with postoperative CHS (*OR* = 5.16, 95% *CI* = 1.09–21.34), which was consistent with previous research [21]. In addition, Zhao M. et al. reported ischemic presentation before surgery was independent risk factors of CHS [14]. Zhang et al. demonstrated hemodynamic source around anastomosis was associated with postoperative CHS, with network formation of MCA origin might be more unstable, and causes greater postoperative hemodynamic changes and more compartmentalized of cortex perfused by each artery than that of non-MCA [12, 31]. In this research, we found that the moyamoya vessel concentration on the surgical hemisphere (*OR* = 3.04, 95% *CI* = 1.02–9.03) was an independent risk factor for CHS after surgery, which had no similar reports before. Heros et al. reported that bypass graft by reversing flow patterns supplied watershed region and induced relative hypoperfusion area distal to anastomosis site [32]. Hayashi et al. reported that dynamic change in cerebral hemodynamics caused by bypass flow could result in the so-called watershed shift at the adjacent cortex to the bypass, which was associated with postoperative transient neurological dysfunctions (TNDs) and cerebral hypoperfusion [30]. According to Poiseuille’s law, vascular resistance is inversely proportional to the 4th power of diameter [33]. Thus, we speculated that these fined, fragile, and dense moyamoya vessels were a huge resistance vessel complex, which was not conductive to watershed shift resulting from new flow pattern after bypass anastomosis. Direct bypass could temporarily lead to heterogeneous hemodynamic distribution on the operated hemisphere due to extensive pial artery network [34]. This abnormal connection between fragile moyamoya vessels and pial vessels in the area of anastomosis could favor the occurrence of postoperative hemorrhage, and dense moyamoya vessels may alter postoperative hemodynamic distribution to be restricted to cortical area, which was detrimental to distribute blood flow from donor artery into deep brain. In addition, Fujimura M. et al. reported that hemodynamic distribution was limited to a relatively small area immediately after the bypass [31]. Thus, we hypothesized that it was the keynote to properly distribute blood flow from bypass grafting into anastomotic vessel network, which was of great value to prevent postoperative hypoperfusion or hyperperfusion. Conversely, it is still unclear how the presence of these moyamoya vessels could be associated with increased perfusion in the cortical area in the absence of hemorrhage. Further evaluation of a larger number of cases with postoperative CHS was warranted to answer this important question.

Moreover, it might be essential to create the criteria of evaluating this deep layer vascular network, namely moyamoya vessel, which was the feature of patients with MMD. In this series, dense moyamoya vessels favored the occurrence of postoperative CHS. Previous literature reported that puff smoke vessels acted as abnormal vascular network formed by collateral pathways to compensate cerebral ischemia, whereas abnormalities of unfused primitive small vessels during arterial development in embryonic and postnatal period were also hypothesized [35]. Piao demonstrated that severe hemodynamic impairment was associated with the extensive development of basal moyamoya vessels for adult patients with ischemic MMD [36]. Zhao Y. reported that the more moyamoya vessels originating from the ICA, the more neovascularization for MMD patients underwent indirect revascularization [18]. We also reported that moyamoya vessels were a sign of brain ischemia and hypoxia, which could alter hemodynamics of thalamus and parietal lobe, with negative correlation for cerebral perfusion and positive correlation for microcirculation parameters [37]. In this study, moyamoya vessels might serve as an important role in surgical alternative options. We speculated that dense moyamoya vessel was a signal of hemispheric perfusion dominant in ICA circulation. Appropriate avoidance of anastomosis with recipient artery connected with dense moyamoya vessels might be beneficial to avoid postoperative CHS. Under this circumstance, indirect bypass overlapping cortex supplied by dense moyamoya vessel might overcome postoperative CHS and achieve more clinical benefit. Storey et al. reported that transdural collaterals dominant in advanced disease are of signal to increased capacity to promote surgical collaterals postoperatively [38]. Consequently, for cases with sparse or none moyamoya vessels in advanced Suzuki stage (5 and 6), hemispheric perfusion supplied by posterior circulation or extracranial circulation was in spontaneous activity predominantly, of which indirect bypass could contribute to neovascularization. Further investigations were needed to understand the role of moyamoya vessels in adult patients with MMD.

Neurological symptoms of CHS were complicated and reversible if treated properly. Cerebral hemorrhage was a disastrous complication in severe cases with the incidence of 3.3~6.6% [9–11]. In this study, 4 patients (2.5%) presented with cerebral hemorrhage, which was consistent with previous reports. Patients with postoperative CHS had higher mean mRS at discharge compared to patients without CHS (1.42 ± 0.52 vs 0.99 ± 0.68, *P* = 0.036). Extensively strict blood pressure control and proper rehydration were particularly important for postoperative CHS, which could balance the risk of hyperperfusion caused by a sudden increase of blood flow and of ischemia caused by insufficient blood supply after surgery. Besides, appropriate use of antiplatelet drugs (aspirin), radical scavengers (edaravone), and matrix metalloproteinase-9 (MMP-9) inhibitors (minocycline) were also able to reduce postoperative CHS [39, 40]. Moreover, Of the 133 patients with average 38-month follow-up, 18 cases (4.91% per person year) had new onset of neurological complications, which was consistent with 0~5.4% per year reported by previous research [41, 42]. There was no significant difference in stroke-free survival and mRS scores between patients with and without CHS. Thus, postoperative CHS could not influence clinical prognosis if treated timely and properly.

This study still had the following limitations. First, the number of MMD patients recruited into research was limited, especially for patients with CHS. The sample size needed to be further expanded. Second, results of this study were from a single center, and multicenter research should be conducted in the future. Third, concentration of moyamoya vessels relied on the experience and perception of radiologist. In the future, black blood sequence of MRI for objective quantification of moyamoya vessels would be useful. Fourth, pre-infarction stage evaluation based on CTP was a semiquantitative method, which could not quantify cerebral perfusion dynamics accurately. The diagnosis of CHS mainly relied on comprehensive analysis of clinical experience and more radiological examinations. The cut-off value of CTP parameters for CHS should be explored in the future. Fifth, absence of intraoperative quantitative hemodynamic measurement, which would be addressed by transit-time ultrasonography combined with FLOW800 or microvascular Doppler ultrasonography (MDU) during surgery.

## Conclusions

The concentration of moyamoya vessels and left operated hemisphere was independent risk factors for CHS, which could not affect the clinical prognosis if treated timely and properly. The current study offers a new perspective of moyamoya vessels and supporting data for choosing MMD candidates on cerebral revascularization.

## Data Availability

The datasets generated during and/or analyzed during the current study are available from the first author on reasonable request (Zhiyong Shi, szy1195156829@aliyun.com).

## Abbreviations

CHS: cerebral hyperperfusion syndrome
STA: superficial temporal artery
EDAS: encephalo-duro-arterio-synangiosis
ICA: internal carotid artery
ACA: anterior cerebral artery
MRA: MR angiography
CTP: CT perfusion
DWI: diffusion-weighted imaging
CBV: cerebral blood volume
CBF: cerebral blood flow
MTT: mean transmit time
TTP: time to peak
TIA: transient ischemic attack
TNDs: transient neurological dysfunctions
